# Global progress, challenges and strategies in eliminating public threat of viral hepatitis

**DOI:** 10.1186/s40249-025-01275-y

**Published:** 2025-02-08

**Authors:** Sihui Zhang, Fuqiang Cui

**Affiliations:** 1 Department of Laboratorial Science and Technology and Vaccine Research Center, School of Public Health, Peking University, Beijing, 100191 China; 2 Center for Infectious Diseases and Policy Research and Global Health and Infectious Diseases Group, Peking University, Beijing, 100191 China; 3 Key Laboratory of Epidemiology of Major Diseases, Ministry of Education (Peking University), Beijing, 100191 China

**Keywords:** Hepatitis B, Hepatitis C, Elimination, Diagnosis, Treatment, Strategy

## Abstract

**Background:**

The problem caused by viral hepatitis is a major public health challenge faced in the past decade, and the global goal of eliminating viral hepatitis by 2030 is still far away. With the use of hepatitis B vaccine and the launch of new drugs, there are more means to control viral hepatitis and more technologies to prevent, diagnose and treat it. While improving the coverage of vaccine use, drugs for treating hepatitis B are not only becoming more effective, but also decreasing in price. The objective of this article was to explore the urgent issues that need to be addressed in global viral hepatitis with the increasing availability of vaccines and antiviral drugs.

**Main text:**

The updated World Health Organization guidelines for the prevention, diagnosis, care and treatment for people with chronic hepatitis B infection (2024 edition) and Chinese guidelines for the prevention and treatment of chronic hepatitis B (version 2022) simplify clinical algorithms for the diagnosis, treatment, and monitoring of hepatitis B, expand treatment eligibility criteria, and provide alternative treatment options, which will cover a higher proportion of all hepatitis B surface antigen positive populations. These actions promote the global goal of eliminating the public health hazards of viral hepatitis by 2030. Among the countries that have made remarkable progress in eliminating viral hepatitis policies, the key strategy is to simplify the diagnosis and treatment plan. Furthermore, the World Health Organization has identified 38 priority countries for viral hepatitis. Expand access to viral hepatitis services in these countries.

**Conclusions:**

Regions and countries with the high burden of viral hepatitis still need to take urgent action regarding the new measures proposed by the WHO to achieve the 2030 targets. First, countries must establish a complete public health system aligned with the World Health Organization’s strategy. Second, provide effective, people-oriented services and public prevention strategies. Third, prioritize the implementation of health strategies in the 38 identified priority countries. Finally, use complete and measurable data to monitor progress.

**Graphical Abstract:**

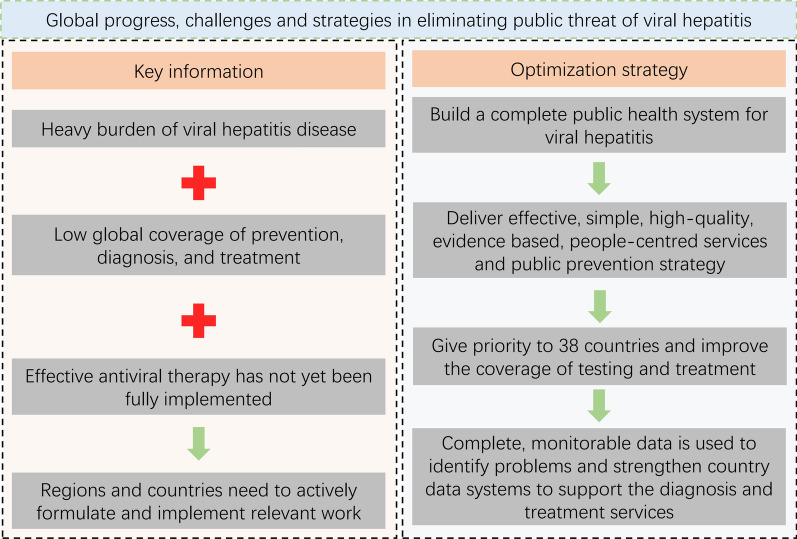

## Background

In the past decade, in order to achieve the World Health Organization (WHO)’s goal of eliminating the public health threats of viral hepatitis, stakeholders of various countries have adopted a series of action plans, mainly by increasing the vaccination coverage for infants and young children to prevent new infections, improving testing and treatment rates to reduce deaths, which have achieved dramatic achievements, but still face enormous challenges. According to estimates from the WHO, viral hepatitis caused about 1.3 million deaths in 2022, including nearly 1.1 million hepatitis B-related deaths and 244,000 hepatitis C-related deaths [1, 2]. The Western Pacific region accounted for 47.0% of the nearly 1.1 million deaths caused by hepatitis B, another 24.7% of deaths occur in Africa, and 19.8% of occur in Southeast Asia, indicating that corresponding measures need to be taken in these regions to reduce the mortality. In 2022, there were 2.2 million new viral hepatitis infections, including 1.2 million new HBV infections and nearly 1.0 million new hepatitis C virus (HCV) infections, compared to 2.5 million new infections in 2019. The decline in incidence highlights the importance of preventive interventions for a sustainable response to viral hepatitis. The African Region and Southeast Asia Region accounted for 62.6 and 21.6% of all new hepatitis B infections, highlighting the importance of expanding access to viral hepatitis services in these two regions and their significance in the global response. In 2016, the 69th World Health Assembly passed the Global Health Sector Strategy on Viral Hepatitis (2016–2021), proposing the goal of eliminating the public health threat of viral hepatitis by 2030, namely to reduce new infections by 90% and deaths by 65%, which means that the baseline number of 6–10 million infections in 2015 need to be reduced to less than 1 million, and 1.4 million deaths reduced to less than 500,000 by 2030 [3, 4]. To achieve this goal, a complete set of strategies and measures need to be developed.

## Challenges and regional variations

Innovative methods are needed to expand the prevention and treatment of hepatitis B and C in different regional and national contexts. Despite achievements in expanding viral hepatitis testing and treatment, global testing and treatment coverage remains low and stagnant. In recent years, many countries have adopted national hepatitis strategies and updated clinical guidelines to improve the accessibility of prevention, testing, and treatment services for viral hepatitis, but their implementation has been lagging. By the end of 2022, only 13% of chronic HBV infected individuals worldwide were diagnosed, and nearly 3% received antiviral treatment, far below the global target. Between 2015 and 2022, only 36% of hepatitis C patients were diagnosed, and 20% received curative treatment, highlighting the need for better linkage between diagnosis and care provision. The diagnosis and treatment rates of hepatitis B in the Western Pacific region were the highest, however, the deaths were also the highest in this region, indicating that further expansion of diagnosis and treatment rates is needed. In African and South-East Asia regions, diagnosis and treatment rates were the lowest, but the deaths account for about 40% of the global, emphasized the provision of diagnostic and treatment services. On the other hand, according to data from 2022 [1], the HBsAg positivity rate was also the highest in the Western Pacific region, followed by Africa and South East Asia regions.

## Countermeasures and strategies

In 2024, the WHO updated its guidelines for the prevention, diagnosis, treatment, and care of individuals infected with chronic hepatitis B [5]. The updated guidelines simplify clinical algorithms for the diagnosis, treatment, and monitoring of hepatitis B, expand treatment eligibility criteria, and provide alternative treatment options. The updated guidelines recommend four treatment eligibility options that will cover a higher proportion (at least 50%) of all HBsAg positive populations, thus providing more opportunities for chronic HBV-infected individuals to receive treatment. China’s 2022 version of the updated chronic hepatitis B prevention and treatment guidelines [6] pointed out that patients over 30 years of age with chronic HBV infection should be treated as long as HBV DNA is positive. Compared with the previous version of the guidelines [7], the restrictions on ALT values were removed, expanding the coverage of treatment for patients with chronic HBV infection. Considering the price of antiviral therapy with nucleoside drugs in China, this measure is also cost-effective [8]. Wong et al. analyzed 89,259 cases of chronic HBV infection with two HBV test results in a laboratory database in the United States. According to the criteria of the American Association for the Study of Liver Disease, European Association for Study of the Liver, Asian Pacific Association for Study of the Liver, and Asian American Treatment Algorithm, the proportions of chronic HBV-infected individuals meeting the treatment criteria were 6.7, 6.2, 5.8, and 16.4%, respectively [9]. But if ALT indicators are removed, the treatment coverage will reach around 87%. However, this strategy should also consider patients’ actual receipt of treatment and their adherence to medication. Therefore, it is also important to focus on doctors’ popularization of patient related knowledge and community education.

Hepatitis B also requires special attention to the African region, where 63% of new hepatitis B infections worldwide occurred, and the diagnosis and treatment coverage of hepatitis B is still below 5%. The same situation also exists in Southeast Asia. In the African region, the diagnosis rate of hepatitis C is still below 15%, and the treatment coverage is below 5%, requiring additional funding and investment to address this issue. The literature has proved that it is cost-effective to screen adults for the universal five indicators of hepatitis B [10]. In these countries and even around the world, chronic hepatitis B infection screening should be expanded, so as to improve the diagnostic rate and achieve simultaneous improvement in the diagnostic rate and treatment coverage. On the other hand, except for the Western Pacific region where the infant birth dose vaccination rate reaches 80%, the timely vaccination coverage of birth dose in other regions is relatively low, particularly in the African region, which has the highest prevalence of hepatitis B. In high-income countries, the burden of hepatitis C among drug users in the United States is increasing. Even among diagnosed patients, treatment coverage remains low, highlighting the urgent need to improve the connection to care.

## Successful experiences for eliminating viral hepatitis

Since 2018, among the 20 countries with the highest burden of viral hepatitis disease globally, 14 countries have made remarkable progress in eliminating viral hepatitis policies, with 5 countries including Bangladesh, India, Indonesia, Japan, and Russia making significant progress. The successful experiences of the above-mentioned countries are summarized as follows: they formulated an action plan to eliminate viral hepatitis; implemented a screening plan; increased the rate of diagnosis and treatment; and provided capital investment, and government subsidies for diagnosis and treatment expenses. The key strategy is to simplify the diagnosis and treatment plan.

## Priority countries

WHO has identified 38 priority countries for viral hepatitis, which collectively account for approximately 80% of the global burden of viral hepatitis. Among them, 10 countries including China, India, Indonesia, Nigeria, Pakistan, Ethiopia, Bangladesh, Vietnam, the Philippines, and the Russian Federation account for almost two-thirds of the total burden. If universal access to prevention, diagnosis, and treatment services is achieved in these countries by 2026, with a focus on supporting work in the African region, it will be a top priority for achieving sustainable development goals globally. Expanding access to viral hepatitis services in these countries will put global response measures back on track to achieve sustainable development goals.

## Conclusions

Regions and countries with the highest burden need to take actions for promoting the global goal of eliminating the public health hazards of viral hepatitis by 2030. First, all countries must take the WHO strategy as the goal in building a complete public health system, develop a national plan to eliminate viral hepatitis, including annual goals, policy support, and key measures. Second, they should deliver effective, simple, high-quality, evidence-based, people-centred services and a public prevention strategy. Third, give priority to the 38 countries and improve the coverage of diagnosis and treatment. Finally, use complete, monitorable data to identify problems and strengthen country data systems to support the diagnosis and treatment services. This is conducive to guiding countries to accelerate the development of data-driven strategies for eliminating viral hepatitis.

## Data Availability

Not applicable.

## Abbreviations

WHO: World Health Organization
HBV: Hepatitis B virus

## References

[1] World Health Organization. Global hepatitis report 2024: action for access in low- and middle-income countries. Geneva: World Health Organization, 2024. https://www.who.int/publications/i/item/9789240091672.

[2] Cui FQ, Blach S, Mingiedi CM, Gonzalez MA, Alaama AS, Mozalevskis A, et al. Global reporting of progress towards elimination of hepatitis B and hepatitis C. Lancet Gastroenterol. 2023;8(4):332–42. 10.1016/S2468-1253(22)00386-7 36764320

[3] World Health Organization. Global health sector strategy on viral hepatitis 2016–2021: towards ending viral hepatitis. https://apps.who.int/iris/bitstream/10665/246177/1/WHO-HIV-2016.06-eng,pdf?ua=1.

[4] World Health Organization. Global Hepatitis Report, 2017. http://www.who.int/hepatitis/publications/global-hepatitis-report2017/en/.

[5] World Health Organization. Guidelines for the prevention, diagnosis, care and treatment for people with chronic hepatitis B infection. Geneva: World Health Organization, 2024. https://www.who.int/publications/i/item/9789240090903. 40424433

[6] You H, Wang FS, Li TS, Xu XY, Sun YM, Nan YM, et al. Guidelines for the prevention and treatment of chronic hepatitis B (version 2022). J Clin Transl Hepato. 2023;11(6):1425–42. 10.14218/JCTH.2023.00320 PMC10500285 37719965

[7] Chinese Society of Infectious Diseases, Chinese Medical Association; Chinese Society of Hepatology, Chinese Medical Association. The guidelines of prevention and treatment for chronic hepatitis B (2019 version). Zhonghua Gan Zang Bing Za Zhi. 2019;27(12):938–61 (In Chinese). 10.3760/cma.j.issn.1007-3418.2019.12.007 PMC12813922 31941257

[8] Zhang SH, Wang C, Liu B, Lu QB, Shang J, Zhou YH, et al. Cost-effectiveness of expanded antiviral treatment for chronic hepatitis B virus infection in China: an economic evaluation. Lancet Reg Health West Pac. 2023;35:10073. 10.1016/j.lanwpc.2023.100738 PMC10326688 37424693

[9] Wong RJ, Kaufman HW, Niles JK, Kapoor H, Gish RG. Simplifying treatment criteria in chronic hepatitis B: reducing barriers to elimination. Clin Infect Dis. 2023;76(3):E791–800. 35594550 10.1093/cid/ciac385

[10] Su S, Wong WC, Zou ZR, Cheng DD, Ong JJ, Chan PL, et al. Cost-effectiveness of universal screening for chronic hepatitis B virus infection in China: an economic evaluation. Lancet Glob Health. 2022;10(2):E278–87. 35063115 10.1016/S2214-109X(21)00517-9 PMC8789560

